# Automated cell lineage reconstruction using label-free 4D microscopy

**DOI:** 10.1093/genetics/iyae135

**Published:** 2024-08-14

**Authors:** Matthew Waliman, Ryan L Johnson, Gunalan Natesan, Neil A Peinado, Shiqin Tan, Anthony Santella, Ray L Hong, Pavak K Shah

**Affiliations:** Department of Electrical and Computer Engineering, University of California, Los Angeles, Los Angeles, CA 90095, USA; Department of Molecular, Cell and Developmental Biology, University of California, Los Angeles, Los Angeles, CA 90095, USA; Department of Molecular, Cell and Developmental Biology, University of California, Los Angeles, Los Angeles, CA 90095, USA; Department of Molecular, Cell and Developmental Biology, University of California, Los Angeles, Los Angeles, CA 90095, USA; Department of Computational and Systems Biology, University of California, Los Angeles, Los Angeles, CA 90095, USA; Molecular Cytology Core, Memorial Sloan Kettering Cancer Center, New York, NY 10065, USA; Department of Biology, California State University, Northridge, Northridge, CA 91325, USA; Department of Molecular, Cell and Developmental Biology, University of California, Los Angeles, Los Angeles, CA 90095, USA; Institute for Quantitative and Computational Biosciences, University of California, Los Angeles, CA 90095, USA

**Keywords:** image analysis, development, cell lineage, nematode

## Abstract

Patterns of lineal descent play a critical role in the development of metazoan embryos. In eutelic organisms that generate a fixed number of somatic cells, invariance in the topology of their cell lineage provides a powerful opportunity to interrogate developmental events with empirical repeatability across individuals. Studies of embryonic development using the nematode *Caenorhabditis elegans* have been drivers of discovery. These studies have depended heavily on high-throughput lineage tracing enabled by 4D fluorescence microscopy and robust computer vision pipelines. For a range of applications, computer-aided yet manual lineage tracing using 4D label-free microscopy remains an essential tool. Deep learning approaches to cell detection and tracking in fluorescence microscopy have advanced significantly in recent years, yet solutions for automating cell detection and tracking in 3D label-free imaging of dense tissues and embryos remain inaccessible. Here, we describe embGAN, a deep learning pipeline that addresses the challenge of automated cell detection and tracking in label-free 3D time-lapse imaging. embGAN requires no manual data annotation for training, learns robust detections that exhibits a high degree of scale invariance, and generalizes well to images acquired in multiple labs on multiple instruments. We characterize embGAN's performance using lineage tracing in the *C. elegans* embryo as a benchmark. embGAN achieves near–state-of-the-art performance in cell detection and tracking, enabling high-throughput studies of cell lineage without the need for fluorescent reporters or transgenics.

## Teaser

Following the pattern of cell divisions in embryos can shed light on essential processes shaping development. While automated cell tracking using fluorescence microscopy is widely available, this requires the generating of transgenic samples. The authors developed embGAN, a software package that can automatically detect cells in 3D imaging using label-free microscopy techniques that don’t require dyes or transgenic samples, making it possible to study the regulation of development in a wider range of animals than previously possible.

## Introduction

Recent advances have led to the development of highly accurate and scalable automated pipelines for reconstructing cell lineages from 3D fluorescence time-lapse images (Santella *et al.* 2010; Wolf *et al.* 2021; Sugawara *et al.* 2022; Malin-Mayor *et al.* 2023). Despite this, manual lineage tracing continues to play an important role in the study of animal development, ever since its use in seminal lineage tracing experiments (Sulston *et al.* 1983). Label-free imaging for small organisms remains more accessible than high-resolution long-term fluorescence imaging, as it avoids the need for transgenesis or staining. Comparative studies in non-model organisms including diverse nematodes (Houthoofd *et al.* 2003, 2006; Houthoofd and Borgonie 2007; Schulze *et al.* 2012) and tardigrades (Hejnol and Schnabel 2005; Heikes *et al.* 2023) have depended heavily on manual tracking using label-free microscopy. Automation, however, has been essential for processing large datasets. Stable transgenic reporter lines in *C. elegans* and well-optimized imaging protocols have facilitated the analysis of several thousands of individual embryos in a variety of contexts (Moore *et al.* 2013; Du *et al.* 2015; Li *et al.* 2019; Cao *et al.* 2020; Ma *et al.* 2021) using automated lineage tracing.

Image analysis approaches have not been extensively developed to detect and track cells in widely used 3D label-free imaging modalities such as differential interference contrast (DIC). Prior efforts based on local entropy and texture detection achieved robust recall but poor precision and were only effective for embryos containing small numbers of nuclei (Yasuda *et al.* 1999; Hamahashi *et al.* 2005). Recently, interest in the analysis of label-free imaging owing to its simplicity and accessibility has motivated the development of deep learning approaches to their manipulation. This includes tools meant for performing computational staining, where a deep neural network is trained to identify subcellular structures in label-free images, and style transfer, where it is trained to transform the appearance of images between imaging modalities (Christiansen *et al.* 2018; Ounkomol *et al.* 2018; Chen *et al.* 2023). These approaches use paired labeled and label-free images of stained or fluorescent samples, minimizing manual generation of training data typically associated with deep learning. These methods have widely used training schemes that minimize pixel-by-pixel measurements of mismatch between the network's output and the real fluorescence modality images provided in the training set. For cell detection and lineage tracing, however, visual similarity is less important than the processed image's compatibility with object detection algorithms.

We developed embGAN to address this gap. embGAN is a deep learning–based pipeline for automated cell detection in 3D label-free imaging. We trained embGAN using 3D fluorescence and DIC imaging of *C. elegans* embryonic development, compared its performance with DIC images relative to a standard lineage tracing pipeline run on matching fluorescence images, and used it to trace the embryonic lineage of wild-type embryos from the widely used laboratory strain N2. We further demonstrate that our pre-trained embGAN model generalizes well to data acquired on different hardware than the training data. embGAN opens the door to automated lineage tracing in situations where fluorescence time lapse imaging is unavailable, where spectral channels for fluorescence need to be conserved for functional reporters, or in genetic backgrounds where transgenesis-induced mutagenesis would be undesirable. To facilitate these applications and continued development, we provide both the trained model, codebase for training and using embGAN, and our complete training image set for use by the community.

## Materials and methods

### *C. elegans* strains and culture

JIM113 was a gift of J. Murray (University of Pennsylvania), and N2 was sourced from the *Caenorhabditis* Genetics Center (University of Minnesota). Strains were grown at 20 ℃ on nematode growth media (US Biological) and fed OP50 *E. coli*. Gravid hermaphrodites from well-fed plates were cut using a needle in M9 buffer and embryos at the 2-cell or early 4-cell stage were mounted using a standard bead-mount approach (Bao and Murray 2011). Briefly, extracted embryos were transferred via a hand-drawn glass capillary to a 2 uL drop of M9 buffer containing ∼100 polystyrene beads 20 um in diameter (Polysciences Inc.), sandwiched between two pieces of #1.5 coverglass, and sealed with melted petrolatum jelly for imaging. RNAi against *cdc-25.1* was performed by feeding the appropriate clone from the Ahringer Lab RNAi collection (Fraser *et al.* 2000). Briefly, RNAi bacteria were seeded onto NGM plates supplemented with 1 mM IPTG and allowed to induce in the dark for 24–48 h. L4 larva were transferred to these plates and allowed to mature and begin producing eggs overnight. The next day, gravid adults were cut and embryos prepped for imaging as above.

### DIC and fluorescence microscopy

Imaging was performed using either DIC alone for N2 or both DIC and fluorescence for JIM113 using an Olympus IX83 inverted frame equipped with a UPLSAPO60xs2 objective, a Visitech iSIM multipoint confocal scanner, ASI MX2000XYZ stage, and Hamamatsu Orca Fusion camera. The mCherry channel of JIM113 was acquired using 594 nm excitation and a 605 nm long-pass emission filter using 150 ms exposures and a laser power that was empirically tuned to not cause any qualitative developmental delays vs un-imaged control embryos and maintain a ∼100% hatch rate for imaged embryos. Embryos were imaged every 60 s with a 750 nm z-spacing. DIC images were acquired with the Visitech scanner in brightfield bypass mode, a 50 ms camera exposure, and the LED light source tuned to not generate any saturated pixels in the image. DIC illumination was generated using an Olympus UCD8 manual condenser equipped with a U525 oil immersion 1.4 NA top lens and a DICTHR tilt-shift slider. The microscope room housing this instrument is maintained at 21 ℃ by forced air cooling, and temperature ranges in the room are checked daily using a thermometer that tracks min/max temperature ranges. Images were acquired using micro-manager and cropped and converted to individual tiff volumes using Fiji. An additional dataset was acquired at a different site (California State University at Northridge) using a Leica DM6000 upright microscope equipped with an HCX PL APO 63x/1.4 NA objective, Leica K5 sCMOS camera, and DIC condenser equipped with a 1.4 NA oil immersion top lens. The DIC slider shear was adjusted empirically to approximate the contrast characteristics of images acquired on our Olympus microscope and images were acquired with a 50 ms exposure, 750 nm z-spacing, and illumination LED intensity adjusted to fill the sensor dynamic range without saturating pixels.

### embGAN

#### Image pre- and postprocessing

Individual volumes were prepared for inference by the embGAN pipeline by performing per-volume contrast adjustment using Fiji's built-in contrast adjustment pipeline set to target a maximum of 0.35% of pixels being saturated. The volumes were then converted to 8 bit grayscale, and individual slices were exported as an image sequence. After inference, the 2D probability maps generated by embGAN were re-assembled into 3D stacks using a Fiji macro. Individual volumes were then clipped at an intensity of −4 by adding 4 to each image and setting all negative pixel values to 0.

#### embGAN implementation and training

embGAN is based on SeGAN, an adversarial generative model developed originally for the task of segmenting partially occluded objects in natural scenes. More specifically, embGAN is an adversarial training framework that utilizes a U-Net as a generator (Segmentor) and a multiscale feature extractor (Critic) as input to a multiscale objective loss function. This loss function is critical to the generalizable performance of embGAN as learning in adversarial networks often exhibits drastic instabilities.

#### Network structure

The segmentor follows a typical U-Net structure, i.e. a convolutional encoder–decoder with skip connections used to connect corresponding levels between the encoder and decoder. The encoder is composed of successive downsampling blocks, each followed by a residual block. The downsample block is comprised of a convolutional layer with stride of 2, each followed by a batchnorm layer and ReLU activation. The decoder mirrors the encoder and takes as input the output of the encoder. Each level of the decoder is made up of an upsampling block followed by a residual block. The upsampling block consists of a bilinear interpolation upsampling layer that performs upsampling by a factor of 2, a convolutional layer, batchnorm, and ReLU activation. The residual block is used in both encoder and decoder and consists of a 1 × 1 convolution, a 3 × 3 convolution, followed by a 1 × 1 convolution. As in the U-Net, we add skip connections between corresponding layers of the encoder and decoder, concatenating the previous encoder outputs to the decoder output.

#### Critic

The critic performs feature extraction on the masked images and is similar in structure to the encoder half of the segmentor without the residual blocks. It also makes use of global convolutions to increase the receptive field while reducing the number of learned parameters. Hierarchical features are computed at each level and are concatenated to produce the final output vector used as input to the multi-scale $l_{MAE}$ loss.

In typical GAN frameworks, the critic network is trained to discern the difference between a ground truth or prediction generated via the segmentor network. To optimize model performance for segmentation, we use the multiscale objective loss function L defined as:


$$min_{\theta_{S}}max_{\theta_{C}}L(\theta_{S},\theta_{C})=\;\frac{1}{N}\overset{N}{\underset{n=1}{\sum }}\;\ell_{mae}(\;f_{C}(x_{n}\circ S(x_{n})),f_{C}(x_{n}\circ y_{n}))$$

where $l_{MAE}$ is the mean absolute error (MAE). The MAE is calculated at multiple scales *n*, between the input image masked by the predicted mask $x_{n}\circ \;S\;(x_{n})$ and the input image masked by the ground-truth labels $x_{n}\circ \;y_{n}$ . $f_{C}$ represents the set of hierarchical features extracted by the critic network from each of the masked images. The $l_{MAE}$ function is defined as:


$$\ell_{mae}(\;f_{C}(x),f_{C}(x^{\prime }))=\frac{1}{L}\overset{L}{\underset{i=1}{\sum }}\;\|\;f_{C}^{i}(x)-f_{C}^{i}\left(x^{\prime }\right){\|}_{1}$$

where *L* is the chosen number of scales in the critic network and $f_{C}^{i}(x)$ is the extracted feature map of image *x* at the ith layer of the critic network.

#### Label generation

Labels were obtained from the fluorescence images using the pre-trained 2D_versatile_fluo model from Stardist.

#### Training

Both segmentor S and critic C networks are trained end-to-end in alternating fashion via backpropagation using a training set containing a total of 112,302 2D images. First, we fix S and train C doing 1 full forward and backward pass and then fix C and do the same for S for 1 step. The process of training S and C resembles a min/max game: S attempts to reduce the multiscale feature loss, while C attempts to increase it. As the training progresses, both S and C networks improve their performance. Eventually, the segmentor can generate high-quality predicted labels that closely match the ground-truth masks. Both networks were trained simultaneously for 10,000 epochs with batch size 36 using the Adam optimizer and a learning rate of 0.0002.

### Automated lineage tracing and performance characterization

embGAN-processed images were processed using the latest release of StarryNite (https://github.com/zhirongbaolab/StarryNite) using a bifurcation classifier model trained using manually curated embGAN data and tuned parameter file (provided in our Github repository: https://github.com/shahlab-ucla/embGAN). For performance characterization, 4 embryos that were imaged separately from the original training set and not included in the training of embGAN were processed both using the fluorescence channel and a parameter file where only the intensity thresholds were adjusted and using embGAN-processed images as input. Curation was performed using the latest release of AceTree (https://github.com/zhirongbaolab/AceTree). All detections in the raw StarryNite output that were not linked to named cells in the curated version were counted as false positives (FPs). Missing cells that were added manually during curation were counted as false negatives (FNs). Errors in tracking were counted as FP if a nucleus was connected to an incorrect successor. We also defined 2 instances of FN for tracking: FNs due to a missing detection and FNs due to a connection that was missed during the tracking stage. For cases with reciprocal errors, such as when tracks swap between 2 cells, previously described as “identity swap” errors, all erroneous edges are counted. In the simple case of 2 cells with swapped identities, a total of 4 errors are counted: 2 FNs for the missing correct edge between each cell and the next time point and 2 FPs for the incorrect edges passing between the tracks.

Tracing the lineages of the amphid neurons was done using AceTree with the embGAN-processed images of JIM113 embryos used in the performance characterization above except that only the sublineages that generate the 2 pairs of 12 amphid neurons on both left and right sides of the animal were edited. Errors were counted using a MATLAB script that compared the pointers in the StarryNite output files connecting detected cells at each time point between the edited and unedited files, and every disagreement was counted as an error. All amphid neurons except for ASE, ASI, ASJ, and ASK were tracked until the birth of the terminal neuron. These 4 were tracked until the birth of the neuroblast 1 cell cycle before the terminal neuron as the terminal neurons are born after an additional round of cell division compared to the remaining 8 amphid neurons. This terminal division often occurs after the beginning of embryonic twitching and thus was excluded from tracking in all embryos for performance characterization. All lineage diagrams were generated using the interactive lineage tool in AceTree and complete lineage diagrams for all embryos presented in this manuscript including both the manually edited trees and each of the initial raw trees generated by StarryNite results, edited only up to the 8-cell stage as required by AceTree to identify the embryo body axes to automate further naming.

### Branch distance comparison of N2 and JIM113 lineages

Whole-embryo and lineage-specific distances were computed using the intersection branch distance (Natesan *et al.* 2023), defined as the *L^2^* norm between a pair of ordered vectors containing the cell cycle times of the corresponding cells that exist in both lineages under comparison. Comparisons between the global clock of development between embryos were performed using the first principal component as a nonparametric measurement of the slope between the cell cycles of matching cells across pairs of embryos under comparison. Comparisons between intra-strain variability based on the median branch distance were performed using the rank sum test in MATLAB r2022a.

## Results

### Pipeline design and model training

**Fig. 1.**
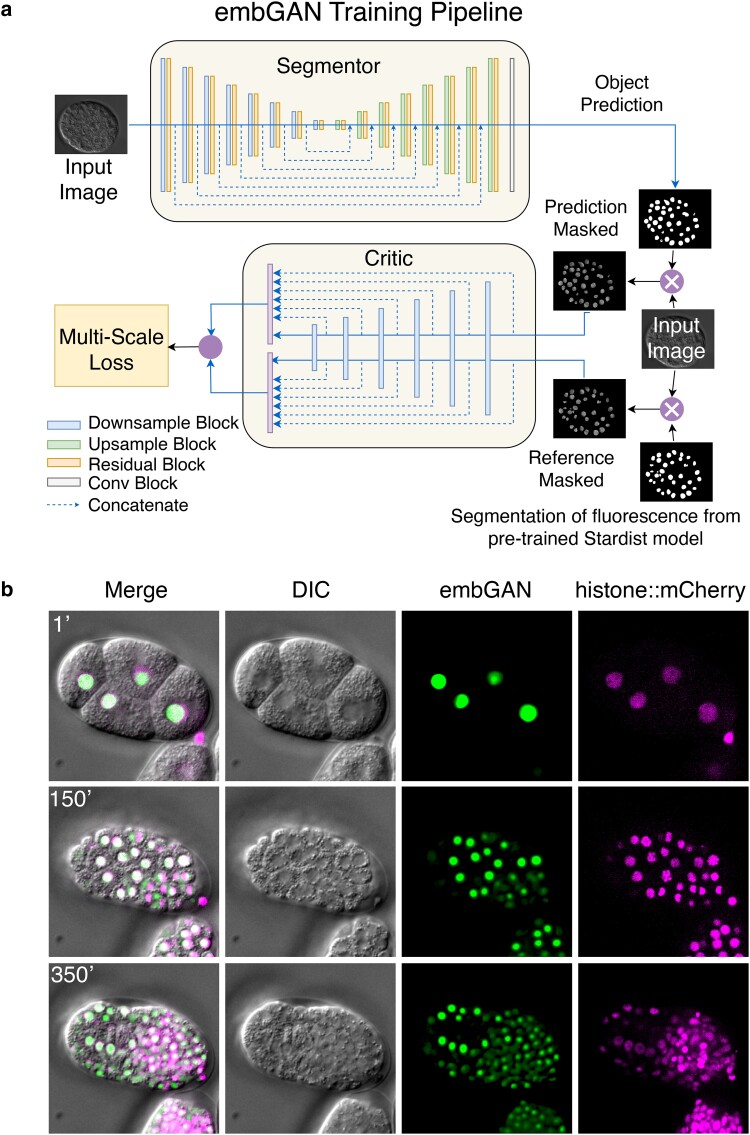
a) Schematic representation of the embGAN network architecture. The Segmentor network generates a segmentation mask of the input image, which is supplied, along with a segmentation generated from the corresponding fluorescence image by a pretrained Stardist model, to the Critic network whose output is used to calculate the multiscale loss which is minimized to train the Segmentor and maximized to train the Critic. b) Example 2D images from the validation image set of a *C. elegans* embryo showing the embGAN probability score (green) along with the input DIC image (gray) and nuclear-localized mCherry (magenta) channel.

The performance and generalizability of deep learning models are heavily influenced by choice of objective loss function. We reasoned that an object-focused loss function that incorporates a segmentation metric would allow a model to better learn the task of cell detection in label-free images given the greater complexity of image features that denote objects of interest, specifically cell nuclei. We thus employed a strategy originally developed for segmenting partially obscured objects in natural scenes (Ehsani *et al.* 2018). In embGAN (Fig. 1a), a U-Net (Ronneberger *et al.* 2015) computes an object probability map from the DIC image to detect cell nuclei. Normally, a segmentation-focused U-Net would be trained using a set of manually labeled training images. Given the variability of DIC image characteristics (i.e. contrast, illumination uniformity, and shadow direction), the wide range of nuclear geometries observed in nematode embryos such as during mitosis and at different stages of development, we aimed to use a training approach not based on manually labeled reference segmentations. Instead, we incorporated a multiscale objective loss function (Xue *et al.* 2018) that uses the output of a second network that serves as a critic of the segmentation. The critic uses imperfect labels generated by a pretrained 2D segmentation model (Schmidt *et al.* 2018) to mask both the input DIC image and the segmentor's output. This strategy allows both the segmentor and critic to learn both object and image properties, ensuring more robust generalization compared to pixel-wise image mismatch-based loss functions.

In our experience, common adversarial training approaches [i.e. pix2pix (Isola *et al.* 2018) or cycleGAN (Zhu *et al.* 2020)] using only fluorescence images and the output of the segmentor as inputs to the critic produced unstable networks that fail to converge or generate reliable predictions on images outside the training set. This brittleness is a well-known challenge with adversarial training (Brock *et al.* 2019), which this loss function addresses by focusing the attention of the critic and thus the loss calculation on object detection performance. We found that even imperfect segmentations by a generalist model dramatically improved the performance and robustness of adversarial training in this task. embGAN was ultimately trained on a dataset containing a total of 112,302 pairs of images in the training set and 41,823 pairs of images in the validation set. These images were acquired using automated time lapse imaging of a total of 11 developing embryos [strain: JIM113 (Zacharias *et al.* 2015)] in both DIC and fluorescence over an average of 35 z-planes per embryo and 6.5 h of imaging (enough to cover from the 2- or 4-cell stage up to the onset of embryonic twitching) to capture the transgenic lineage tracing reporter. We employed a probabilistic strategy where the U-Net generates an output image with continuously varying values rather than a binary segmentation. This output format makes images generated by embGAN compatible with a wide range of downstream cell detection and tracking pipelines developed for use with fluorescence microscopy. Tuning parameters used in these algorithms for processing embGAN images then allows end users to optimize performance for their task, for example, by reducing FNs at the cost of a potential increase in FPs by reducing cell detection thresholds.

### embGAN robustly reconstructs the cell lineage of the early *C. elegans* embryo

We tested embGAN performance on a lineage tracing task with a well-characterized ground-truth: reconstructing the *C. elegans* (Sulston *et al.* 1983) embryonic cell lineage. We acquired test data of JIM113 embryos separate from the training set using time lapse 3D microscopy. We measured real-world cell detection and tracking performance using StarryNite (Santella *et al.* 2010), a well-characterized and robust cell detection and tracking pipeline. After training a new classifier model for StarryNite's tracking pipeline using a manually curated dataset, we optimized StarryNite performance for processing embGAN images solely by adjusting the intensity thresholds and cell diameter limits applied to StarryNite's difference-of-Gaussians filter for cell detection. No other tracking or detection parameters were adjusted from defaults provided by the Bao Lab (https://github.com/shahlab-ucla/embGAN) along with the StarryNite codebase.

**Fig. 2.**
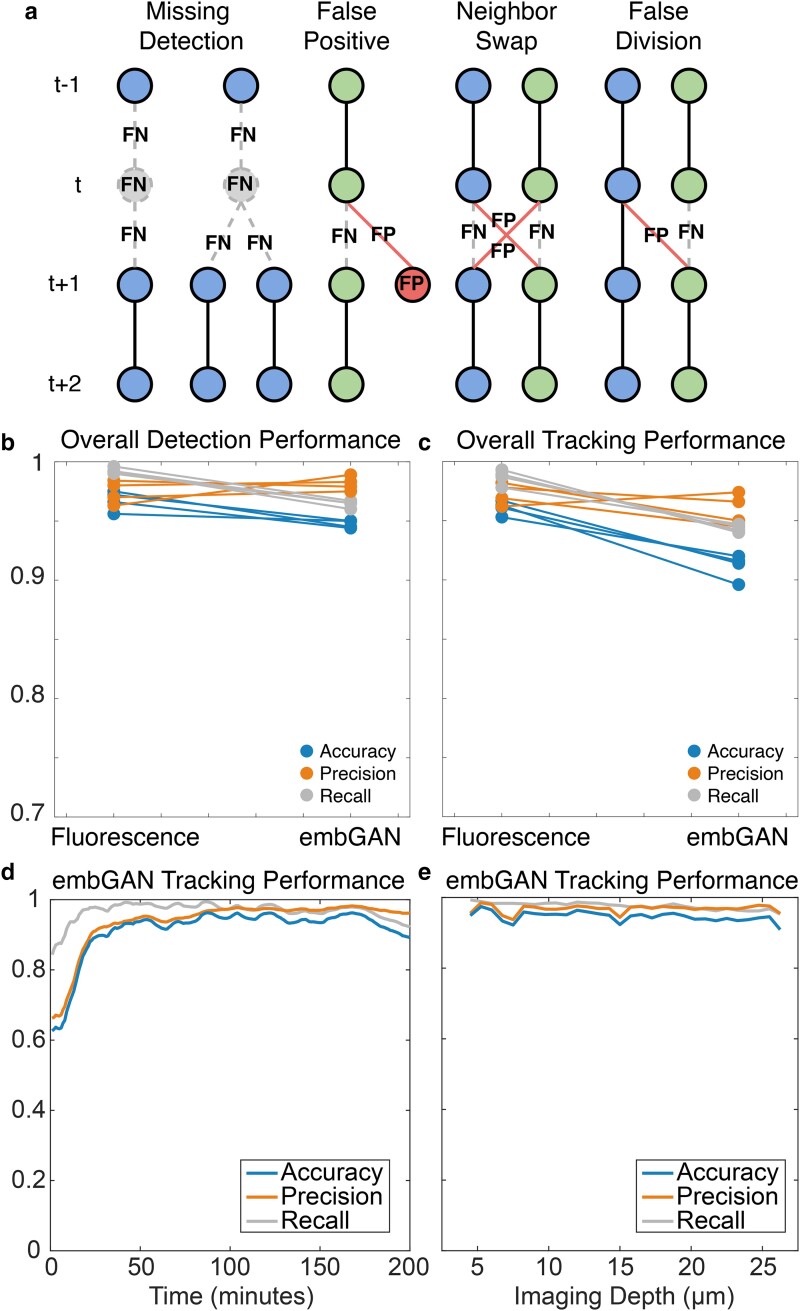
a) Schematics of common detection and tracking errors. Circles represent cell detections; gray circles show FN and red circles show FP. Lines show tracked edges between detections across time points. Dashed lines show FN edges and red lines show FP edges. b) Detection and c) tracking performance measured on 4 embryos. Each point shows the measurement of accuracy (blue), precision (orange), and recall (gray). Lines connect measurements between StarryNite performance on fluorescence imaging (left) or embGAN-processed DIC imaging (right) of the same embryo. Tracking performance in embGAN images as a function of d) developmental time and e) axial position within the image volume. Accuracy (blue), precision (orange), and recall (gray) shown as in b) and c), with the performance over time smoothed using a 10-frame moving average.

We separately processed the fluorescence time lapse and DIC images processed by embGAN for 4 embryos with StarryNite (Fig. 1b). We then manually curated both sets of results from all 4 embryos for the first 200 min of development (a total of 1,600 volumes). Comparing the automated results against manual curations, we counted all detection (Supplementary Tables 1 and 2; Supplementary Figures 1 and 2) and tracking (Supplementary Tables 3 and 4; Supplementary Figures 3 and 4) errors (Fig. 2a) in each dataset. StarryNite performs extremely well on cell detection tasks with fluorescence images (Fig. 2b), with recall exceeding 99% (99% of all cells are detected) and precision averaging 97.4% (2.6% of detections are FPs). For tracking, StarryNite performance on fluorescence images achieved recall and precision averaging 99 and 95.9% (Fig. 2c), respectively. Since embGAN is trained using a critic fed with imperfect segmentations, StarryNite cell detection and tracking performance drops slightly compared to corresponding fluorescence images. Despite this, embGAN performance averaged 98.2% recall and 96.4% precision for detection (Fig. 2b) and averaged 95.9% recall and 94.4% precision for tracking (Fig. 2c).

To clarify the strengths and weaknesses of using embGAN-generated images for lineage tracing with StarryNite we characterized tracking performance as a function of time (Fig. 2d) and axial position within the embryo (Fig. 2e). Tracking performance at early time points appears to vary wildly, as the small number of cells per-frame results in large fluctuations of these metrics. Overall tracking performance stabilizes and remains consistent on a per-frame basis over the period of development assessed (the first 200 min of proliferation in the embryo) and is similarly consistent as a function of axial depth. We further dissected tracking and detection performance in terms of absolute true positive and error rates on a per-embryo basis for lineage tracing performed in fluorescence images and in embGAN-processed DIC images (Supplementary Figs. 1–4). In general, StarryNite detection errors on embGAN images occur most during periods of development where large numbers of cell divisions are occurring. Detection errors are somewhat correlated with increasing depth into the sample, although this seems to vary from embryo to embryo, which generally matches trends seen in fluorescence imaging where increasing depth results in signal attenuation due to scattering, absorption, and depth-dependent aberrations.

As would be expected given StarryNite's tracking approach being based on greedy linking to nearby detections, most FNs in tracking are caused by detection errors. Also similar to StarryNite’s performance on fluorescence images, both FP and FN tracking errors are similarly correlated to periods of development when cell divisions are occurring. Tracking errors independent of detection errors are uniformly distributed along the depth of the embryo. Examination of embGAN prediction confidence during cell divisions shows that these detection and related tracking errors are caused by a drop in confidence during cytokinesis, visualized as the intensity of the embGAN image channel (Supplementary Movie S1). Future refinement of the masking step used in training embGAN or enrichment of cell divisions in the training set may further improve embGAN performance during cell divisions in the future. Complete lineage diagrams of each of these 5 embryos are provided in Supplementary File S1, including the fully edited diagrams for each embryo and the initial output of StarryNite for both fluorescence and embGAN images edited only up to the point necessary for AceTree to establish cell naming based on the convention established by Sulston *et al.* (Sulston *et al.* 1983).

### embGAN performs well in lineage tracing tasks spanning late embryogenesis

**Fig. 3.**
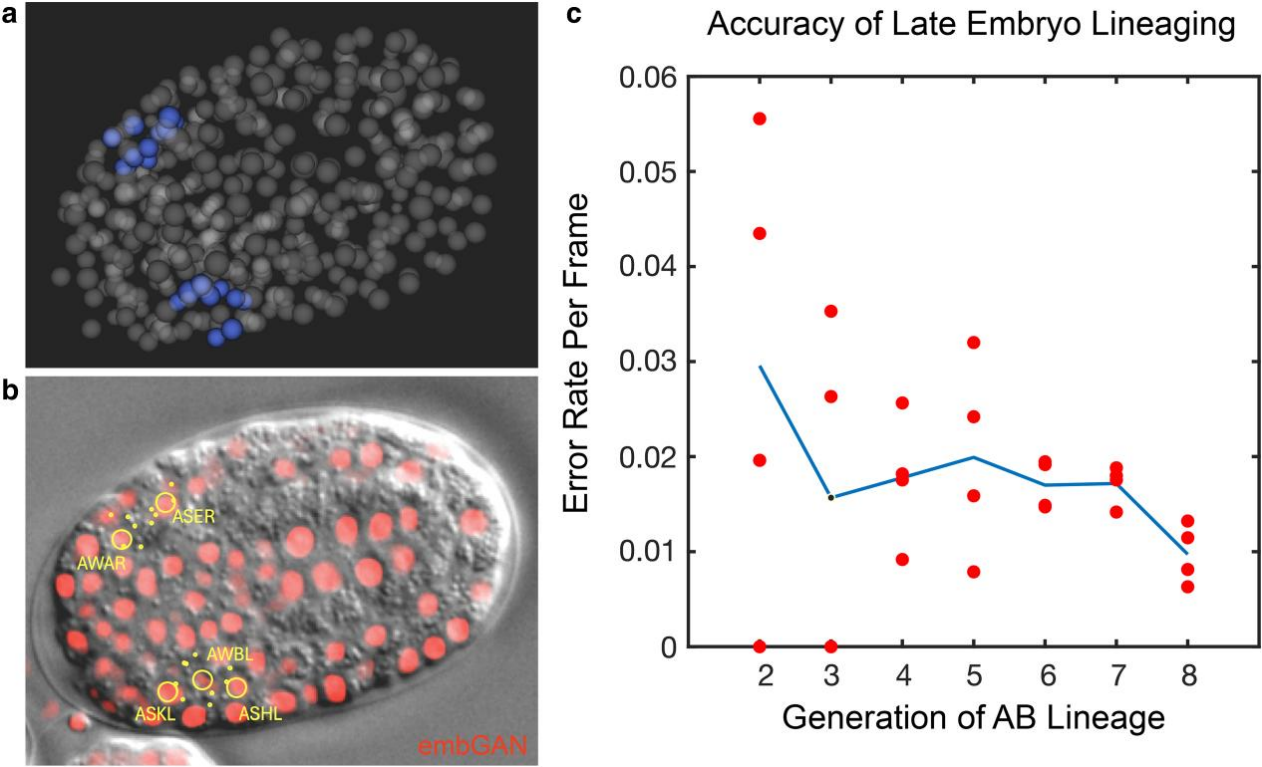
a) 3D rendering of amphid cell positions traced using embGAN images. b) Image of DIC and embGAN (red) image with annotations showing positions of amphid cells. Cells present at the focal plane are circled with dots showing the XY position of cells present at different depths in the volume. c) Accuracy of lineaging cells born late in embryogenesis. Mean (blue line) and per-embryo (red circles) error rates for lineaging amphid neurons in embGAN processed images.

Since embGAN performed well for lineage reconstruction in the early embryo, we next tested it at later stages of development when cell nuclei are much smaller and more densely packed, often with no separation between adjacent nuclei. Since embGAN performance remained stable up until 200 min of development as evaluated embryo-wide (Fig. 2d), we expect the total number of errors to scale into later stages of development as the total number of cells in the embryo increases and the number of cell divisions continues to increase as well. Thus, to evaluate embGAN's suitability to a range of lineage tracing tasks requiring identifying terminally divided cells in the late embryo, we tracked the 24 neurons of the amphid sensory organs in the 4 test embryos. These neurons are born in either the final 9th or 10th generations of the AB lineage (Fig. 3a and b). We tracked the 8 neurons born in the 10th generation up until the birth of their parents, since their terminal division sometimes occurs after the beginning of rapid embryo movement, which prevents reliable tracking using StarryNite. We counted all tracking errors in the lineage history of these cells, finding that most errors occur due to cell divisions, either the cell's own or a nearby cell's, such that a similar proportion of errors occurred in each generation of the AB lineage. Since the length of the cell cycle of each generation also increases, this results in a decrease in the per-frame error rate at later generations compared to earlier generations starting at 3% in the early embryo and dropping to ∼1% (Fig. 3c).

### embGAN generalizes well to new strains, images from different microscopes, and cell fate transformations

**Fig. 4.**
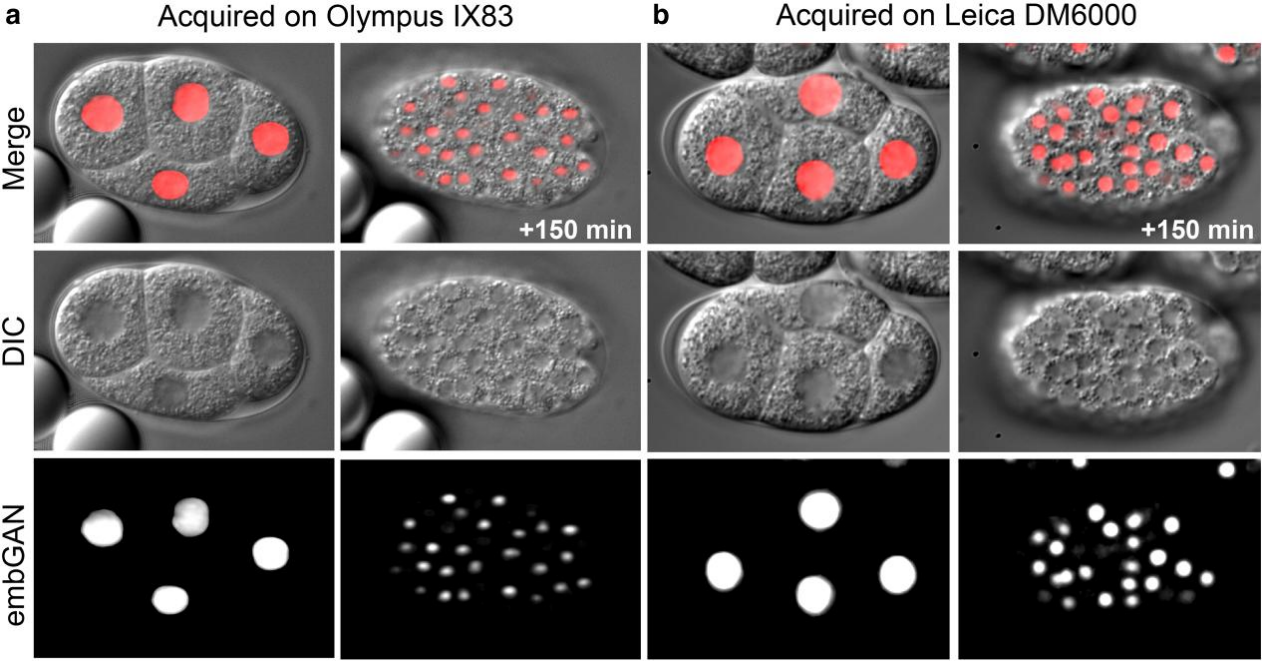
a) Generalizability of embGAN model across multiple source microscopes. Images of early and mid-stage N2 embryos shown in DIC, embGAN output, and merged channels acquired on the same microscope as the embGAN training data (Olympus IX83) and b) a second microscope using different hardware (Leica DM6000).

**Fig. 5.**
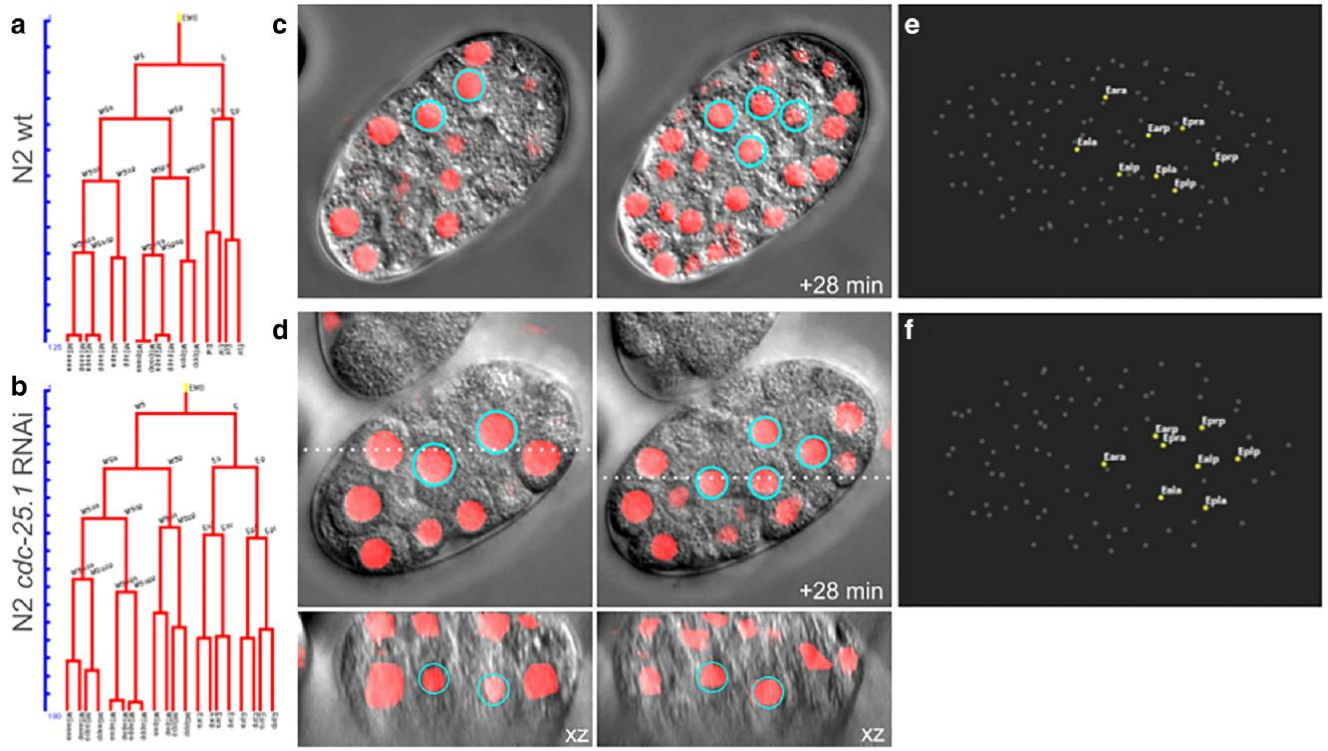
Generalizability of embGAN model to lineage tracing in genetically perturbed embryos. Lineage diagrams of the EMS lineage in a) wild-type N2 and b) *cdc-25.1* RNAi-treated embryos. The vertical scale shows time elapsed in minutes. DIC (gray) and embGAN predictions (red) of the E2 and E4 stages when E cells (cyan circles) normally gastrulate in c) wild-type and d) *cdc-25.1* RNAi-treated embryos. Insets show xz-axis slices through the volumes at the position marked by dash lines showing no evidence of gastrulation in *cdc-25.1* RNAi embryos. 3D renderings of E8 cell positions in e) wild-type and d) *cdc-25.1* RNAi-treated embryos.

We next tested embGAN's ability generalize to images of animals from different strains and to images acquired on a different microscope than the training set. For this, we imaged the laboratory-raised wild-type strain N2 and applied embGAN to these images (Fig. 4a). We imaged N2 embryos using 2 different brands of microscope (Leica DM6000 vs Olympus IX83), different objectives and immersion media (63× oil vs 60× silicone oil immersion), and different cameras (Leica K5 vs Hamamatsu C14440-20UP). Both sets of images, including those acquired of N2 embryos on the second microscope show excellent correspondence between the predicted location of nuclei and observed nuclei in the DIC images (Fig. 4b). Based on these results, we set out to use embGAN to perform a quantitative comparison of embryonic cell cycle timing in the canonical laboratory wild-type strain N2 and JIM113, a commonly used transgenic strain for lineage tracing by fluorescence imaging. Complete lineage diagrams of all N2 and JIM113 embryos analyzed here are provided in Supplementary File S1, including the fully edited diagrams for each embryo and the initial, raw output of StarryNite for each embryo. We further tested the generalizability of our embGAN model by applying it to the analysis of an abnormal lineage phenotype by characterizing the effects of *cdc-25.1* RNAi on the specification of the E lineage. *cdc-25.1* encodes a CDK phosphatase that promotes cell cycle progression by removing inhibitory phosphates. Loss of *cdc-25.1* expression is known to cause loss of endodermal fate in the E lineage (Du *et al.* 2015). The lineage of an RNAi-treated embryo shows an overall increase in cell cycle duration, changes in the structure of the MSp lineage, and dramatic shortening of cell cycle durations in the E lineage (Fig. 5a and b). The E lineage normally undergoes gastrulation at the E2 stage (Lee and Goldstein 2003). In the orientation shown (Fig. 5c), gastrulation occurs in-plane with the imaging. Disruption of E's fate due to *cdc-25.1* RNAi prevents gastrulation with the E4 cells remaining at the embryo surface as apparent in the lateral and transverse slices (Fig. 5d). In wild-type embryos, the E8 stage normally produces a clear 2 × 4 planar arrangement of cells (Fig. 5e) but the 3D organization of this tissue is also lost in *cdc-25.1* RNAi (Fig. 5f).

### N2 embryos exhibit greater inter-embryo variability in cell cycle duration

**Fig. 6.**
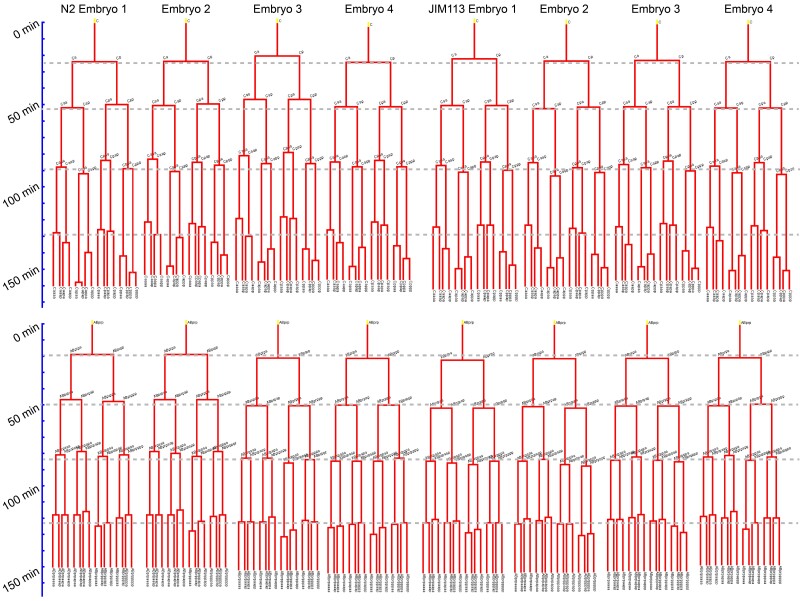
Tree diagrams of the C and ABprp lineages sampled from 4 examples of N2 and JIM113 lineages. Each tree is scaled uniformly, and gray dashed lines are provided for visual reference to compare cell cycle consistency across lineages.

**Fig. 7.**
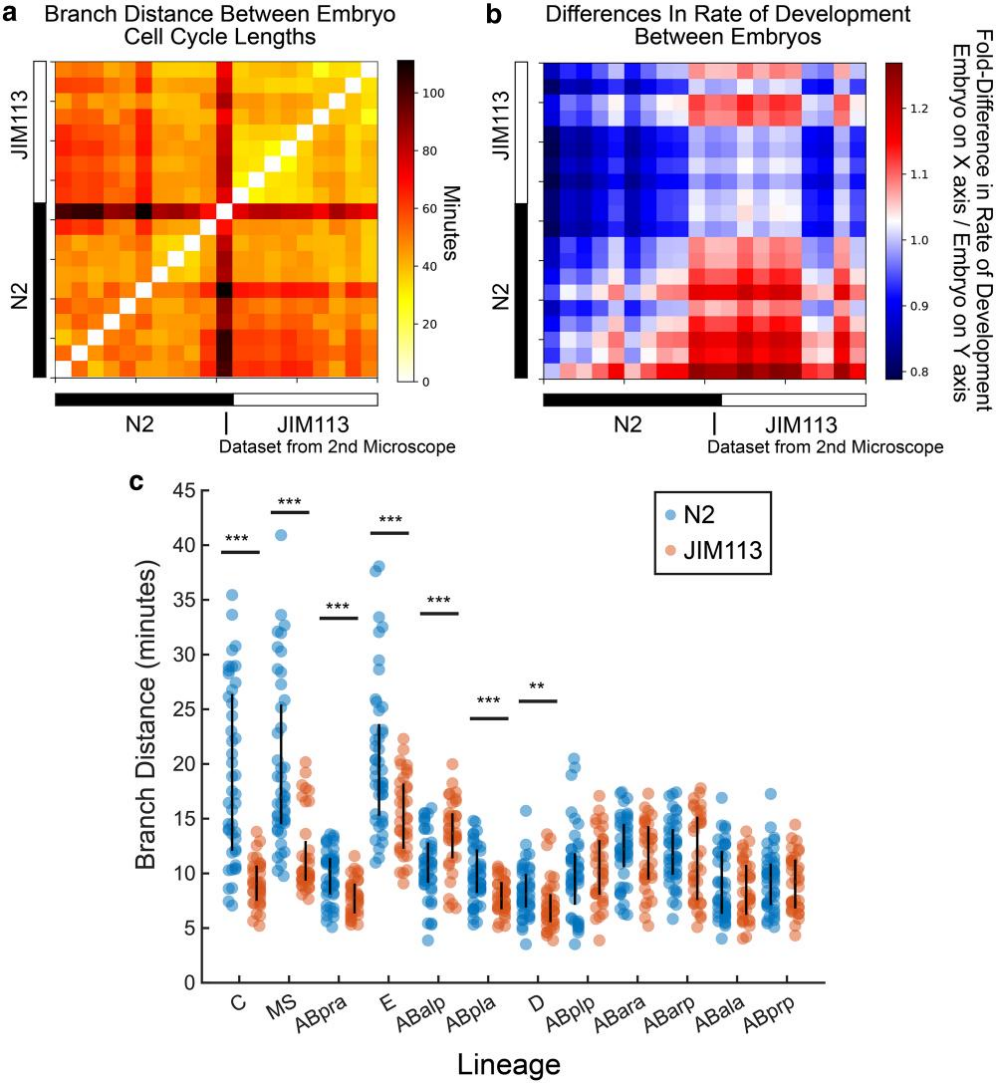
a) Heatmap showing the pairwise branch distance comparing lineage-aligned cell cycle lengths between all N2 and JIM113 embryos. The single N2 embryo imaged using the second microscope is highlighted at the boundary between N2 and JIM113 embryos. b) Comparison of global rate of development between all N2 (black bar) and JIM113 (white bar) embryos calculated pairwise using the first principal component as a nonparametric estimate of scaling between embryos. Each square represents a single pairwise comparison where the color reflects the fold-difference in cell cycle durations of the embryo indicated by the x-coordinate of the square relative to the embryo indicated by the y-coordinate of the square. c) Distribution of pairwise branch distances between specific lineages within N2 (blue) and JIM113 (orange) embryos. Vertical black line shows the interquartile range. ***P < 0.001 and **P < 0.01 by the rank sum test.

Using embGAN and StarryNite, we tracked all cells through the 6th round of division of the AB blastomere and a similar corresponding stage for all other sublineages of the embryo in 10 N2 embryos imaged using the Olympus microscope and one of the N2 embryos imaged using the Leica microscope. Lineages for each embryo were reconstructed using AceTree (Katzman *et al.* 2018; Fig. 6). We used the branch distance, a metric we previously developed to compare lineage-aligned phenotypic measurements between cell lineages (Natesan *et al.* 2023), as a summary statistic to compare the lineage-aligned distribution of cell cycle durations between the same cells in N2 and a set of transgenic JIM113 embryos (Fig. 7a). The embryo imaged using the Leica microscope was a clear outlier here due to a difference in ambient temperature during imaging (∼23 ℃ vs 21 ℃) causing a change in the global rate of development (Fig. 7b).

Interestingly, N2 embryos exhibited ∼2× more intrastrain variability in the branch distance than JIM113 embryos. This increased variability reflects differences in cell cycle timing between N2 embryos and originates consistently from specific lineages (Fig. 7c). Examination of lineage trees show that this increased variability originates from diverse individual cells within these lineages but predominantly from later-born generations. N2 embryos are more heterogeneous than JIM113 among all lineages with significant interstrain differences in the median branch distance between embryos of the same strain except for 1—ABalp. Previous studies of cell division timing consistency of N2 embryos using manual lineage tracing noted a similar variability (Schnabel *et al.* 1997), and our comparisons with JIM113 highlights interesting but as yet unexplained differences in timing variation between this transgenic strain derived from N2 and N2 itself.

## Discussion

Automated pipelines for cell detection and tracking in 3D time-lapse fluorescence microscopy have advanced by leaps and bounds in recent years. These efforts have principally focused on improving performance on image sets of organisms too large to generate large amounts of manual annotation for deep learning (Sugawara *et al.* 2022; Malin-Mayor *et al.* 2023). Label-free imaging remains an attractive technology for the study of early development as it does not require transgenic or stained samples. The approach we employ in embGAN makes it possible to achieve the performance required for high-throughput automated lineage tracing using label-free images. The multiscale loss function we adopted in embGAN based on prior work (Xue *et al.* 2018) allows us to combine the widely characterized advantages of adversarial training using discriminative features that are learned by the critic network during the training process with the attention-focusing effects of limiting both the input and segmentation predictions to regions of the image containing cell nuclei using segmentation masks generated from the fluorescence channel acquired in the training dataset.

We anticipate that this strategy could be extended in the future using multiple strategies to better capture different features of cellular morphology, for example, by generating masks based on 3D segmentations of cell morphology and not just the nucleus (Cao *et al.* 2020). Using higher information content imaging modalities such as quantitative phase imaging (QPI), larger and more diverse training sets, or higher performance fluorescence segmentation models for training data annotation will likely further improve embGAN performance. We prototype a novel potential application for embGAN as a tool for comparative embryology between strains of distinct genetic backgrounds.

Despite the penalty in detection and tracking accuracy compared to the use of fluorescence images, embGAN achieves tracking robust enough to perform a wide range of common lineage tracing tasks. From our experience using embGAN for lineage tracing thus far, we estimate that the reduced accuracy carries a qualitative effort burden of ∼2.5× in terms of the time required to manually curate the automated results produced by StarryNite as compared to a similarly experienced user working with fluorescence images. Perhaps a more salient comparison, starting from embGAN and StarryNite-generated lineages and manually correcting errors results in a 95% or more reduction (in other words, a ≥20× difference) in the total number of user interventions required to produce an empirically accurate lineage relative to fully manual lineage tracing (estimated based on the rate of errors that would require correction as a proportion of the total number of expected cells per frame).

This proportion is a conservative estimate as many individual edits would simultaneously correct 2 lineage errors as we have classified them. Over the test set, 64% of tracking errors were caused by detection errors, suggesting that future improvements in embGAN prediction performance is the most promising route toward more robust overall performance. Other methods to attempt to improve tracking performance, such as increasing temporal sampling, is unlikely to have a dramatic impact on performance as frame-to-frame cell movement is generally much smaller than a nuclear radius except for during cell divisions. The decreased accuracy of embGAN in detecting cells during cell divisions is most likely caused by a mismatch during training between the geometry of the segmentation mask generated from histone fluorescence (compact pronuclei) and the informative features of DIC images during cytokinesis (a larger swatch of the cell, locally smooth in texture and without a sharp boundary).

As a proof-of-concept of the potential utility of automated lineage tracing in nontransgenic *C. elegans* embryos, we traced the early lineage of N2 embryos, something previously only possible using fully manual approaches (Schnabel *et al.* 1997). We further examined the generalizability of embGAN by tracing the lineage of N2 embryos treated with RNAi against *cdc-25.1*, a CDK phosphatase that plays a major role in cell cycle regulation. Loss of *cdc-25.1* results in an average increase in cell cycle duration and is known to induce dramatic cell fate transformations, one of which results in the E lineage losing its endodermal identity. This transformation shortens the cell cycles of the E lineage and blocks its gastrulation, disrupting the organization of the embryo. Despite this drastic change in the spatial distribution of cells within the embryo, embGAN can reliably identify cell nuclei and enable the identification of the lineage transformation caused by RNAi treatment.

As a proof-of-concept for the characterization of interstrain differences using embGAN, we thought to compare the topology of N2 lineages against those produced for JIM113, a strain that has become established as a workhorse for automated lineage tracing by fluorescence microscopy. We were surprised to note dramatic differences in the consistency of cell cycle timing between these strains and that these differences themselves appear patterned such that more posterior lineages in the embryo (MS, E, C, D, and many posterior AB-derived lineages) exhibit both statistically and qualitatively clear differences in variability between the strains. This general observation, that N2 exhibits clear variability in cell cycle timing in many lineages, is consistent with prior studies (Schnabel *et al.* 1997), but an explanation for the lack of such variability in JIM113 remains unclear. JIM113 is itself a transgenic strain derived from N2 via biolistic bombardment; it was likely passaged through single worm population bottlenecks for several generations during the selection process and was subsequently outcrossed against N2 for 2 generations, which would have necessitated further passaging by picking single worms. Whether this type of population bottleneck could have such a homogenizing effect on developmental variability and whether this is somehow specific to the N2 genetic background or might be a general phenomenon across diverse *C. elegans* genotypes might warrant future investigations into the origins of variability in the dynamics of embryogenesis.

embGAN makes high-throughput cell lineage analysis possible for a wider range of sample types than was previously possible and introduces useful experimental flexibility by removing the need for a fluorescent marker for semi-automated lineage tracing. It generalizes well across *C. elegans* strains and imaging conditions.

## Data Availability

To accelerate continued improvements in this area, we are making our full training image set freely available (https://datadryad.org, DOI: 10.5061/dryad.zcrjdfnkz) alongside the codebase and trained model weights for embGAN (https://github.com/shahlab-ucla/embGAN; DOI: 10.5281/zenodo.10535870). Supplementary Material is available via Figshare https://doi.org/10.25386/genetics.25979614.
